# The statistical shape model as a quality assurance measure in the treatment of complex midface fractures: a case control study

**DOI:** 10.1186/s13005-021-00296-w

**Published:** 2021-10-20

**Authors:** Marc Anton Fuessinger, Steffen Schwarz, Mathieu Gass, Philipp Poxleitner, Leonard Brandenburg, Stefan Schlager, Marc Christian Metzger

**Affiliations:** 1 Department of Oral and Maxillofacial Surgery, Albert-Ludwigs University Freiburg, Hugstetterstr. 55, 79106 Freiburg, Germany; 2 Department of Physical Anthropology, Albert-Ludwigs University Freiburg, Hebelstr. 29, 79104 Freiburg, Germany

**Keywords:** Statistical shape model (SSM), Computer-assisted surgery (CAS), Virtual defect reconstruction, 3D planning, Virtual planning, Bilateral midface fracture

## Abstract

**Background:**

Complex bilateral midface fractures necessitate a surgically challenging procedure to preserve or restore the occlusion and the sensitive eye area. In this case control study, we aim to show the potential of a statistical shape model (SSM) for measuring the quality of the midface reconstruction, compared to the estimated preoperative situation.

**Methods:**

An individualized SSM was postoperatively registered on 19 reconstructed complex bilateral midface fractures. Using this SSM, the distances from the simulated preoperative situation to the postoperative positions of the fracture segments were calculated. The fracture lines for Le Fort II, Le Fort III, and NOE fractures were chosen as reference points for the distance measurements.

**Results:**

The SSM could be registered on all 19 complex bilateral midface fractures. All analyzed fractures showed a dorsal impaction (negative values) of the midface. Le Fort II fractures showed deviation values of –0.98 ± 4.6 mm, Le Fort III fractures showed values of –3.68 ± 3.6 mm, NOE type 2 fractures showed values of –0.25 ± 4.6 mm, and NOE type 1 fractures showed values of –0.25 ± 4.6 mm.

**Conclusions:**

The SSM can be used to measure the quality of the achieved reduction of complex bilateral midface fractures based on the estimated preoperative situation.

**Trial registration:**

DRKS00009719.

## Introduction

The epidemiology of midfacial fractures depends on the geographical areas and socio-economic statuses of the considered countries [1–3]. In more than 50 % of the cases, the zygomaticomaxillary complex is the most common unilateral fracture site [4]. Irrespective of the affected anatomical region, traffic accidents are the most common cause in western countries. In less economically advanced countries, interpersonal violence like fights, assaults, and gunshots are frequent reasons for midface fractures [4]. The presence of a bilateral midface fracture is associated with high-velocity trauma, caused by, e.g., road traffic accidents, jumps in suicidal intent, or work-related accidents [5]. In most cases, the structure, function, and the appearance of the midface, including the globe, are altered by the midfacial fractures [4, 6, 7]. Restoration of the function and form of the face by respecting the original facial width, facial height, and facial projection aims at preventing long-term complications and enabling the patient’s re-integration into everyday life [4, 8, 9].

Helpful tools for the precise restauration of midface fractures are up-to-date preoperative imaging modalities, like magnetic resonance imaging (MRI) or computed tomography (CT), and intraoperative tools, like imaging (cone beam CT (CBCT)) and navigation by using computer-assisted surgery (CAS) for complex trauma, oncologic resections, and reconstructions and congenital deformity cases [10]. For unilateral fractures, CAS is well established [11]. The preoperative planning of the restoration or manufacturing of patient-specific-implants (PIS) is done by mirroring as the “gold standard”. More advanced techniques are in development [12]. As one opportunity, we show the advantages of a statistical shape model (SSM) in preoperative virtual planning for repairing artificial unilateral midface and neurocranium defects [13, 14]. Artificial bilateral defects of the midface could also be reconstructed satisfactorily [5]. The purpose of this study is to demonstrate the application of the SSM for measuring the quality of the postoperative result in the reconstruction of bilateral midface fractures. The possibility to register the individualized SSM on the patient skull is one aim of this study. A second aim is to calculate the distances between the achieved results of the midface fracture reduction and the proposed position of the midface, using the SSM.

## Materials and methods

To address the research purpose, the investigators designed and implemented a retrospective case control study. The clinically achieved reposition served as control. The virtually reconstructed midfaces using the SSM serves as test group. Ethical approval of the Ethical Review Board of the University Hospital Freiburg according to the Declaration of Helsinki on medical protocols and ethics was obtained.

The study population comprised all patients having presented with complex bilateral midfacial fractures in our clinic, between January 2014 and January 2019.

To be included in the study sample, patients had to show bilateral midface fractures that had been treated by surgical fracture reduction and fixation. Missing pre- or postoperative CT imaging and inadequate imaging quality were defined as exclusion criteria. The preoperative CT scans were inspected and grouped into typical fracture patterns according to the present AO classification.

The core of the proposed method for postoperative determination of the reconstruction quality consists in an SSM generated from healthy patient data, in order to capture the three-dimensional shape variability of the cranium in healthy European adults. SSMs are employed in a variety of applications to generate valid shapes from noisy or partial data.

In our case, it is used to find the shape that best matches the partial (i.e. defective) cranial shape.

The SSM used in this study was generated based on 131 craniofacial CT scans of patients without cranial injury or defect (n = 61 female, n = 70 male patients; average age = 53.2 years). The slice thickness of the CT scans ranged from 1 to 2 mm.

The study data consists of postoperative images generated from routinely performed diagnostic trauma CT scans of the midface region. A 128-row multi-slice CT scanner (Somatom Definition Flash; Siemens, Erlangen, Germany) was used to obtain the CT scans. A slice thickness of at least 1 mm was required. The data were exported as DICOM files from the PACS system. The files were imported into 3D Slicer. Threshold segmentation of the DICOM files was done using 3D Slicer (slicer.org). The achieved triangulated surface data of the midface region were stored in the .ply file format.

**Fig. 1:**
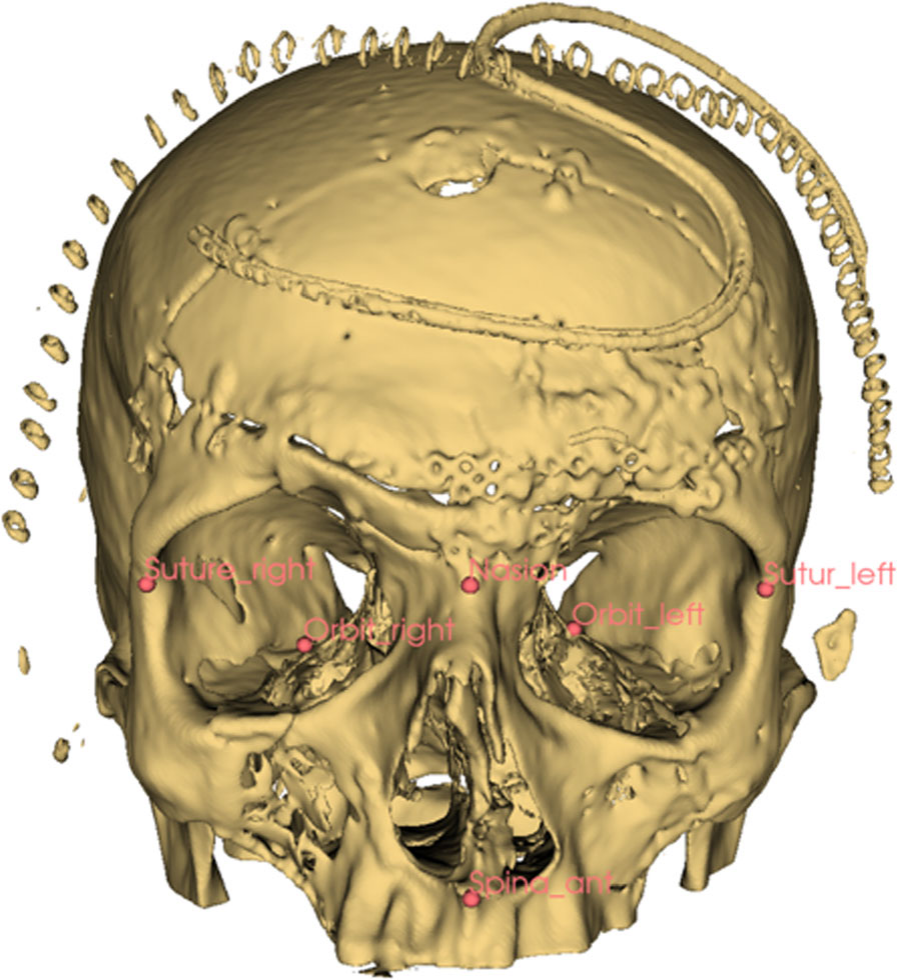
Example of a Le Fort II fracture after repositioning and osteosynthesis. The landmarks for alignment of the SSM on the patient’s skull are shown as red dots

In 3D Slicer, fiducials are set as landmarks. Landmarks are the bony external acoustic meatus, the zygomaticofrontal suture, the nasion, the anterior nasal spine, and the posterior orbital floor (Fig. 1).

**Fig. 2:**
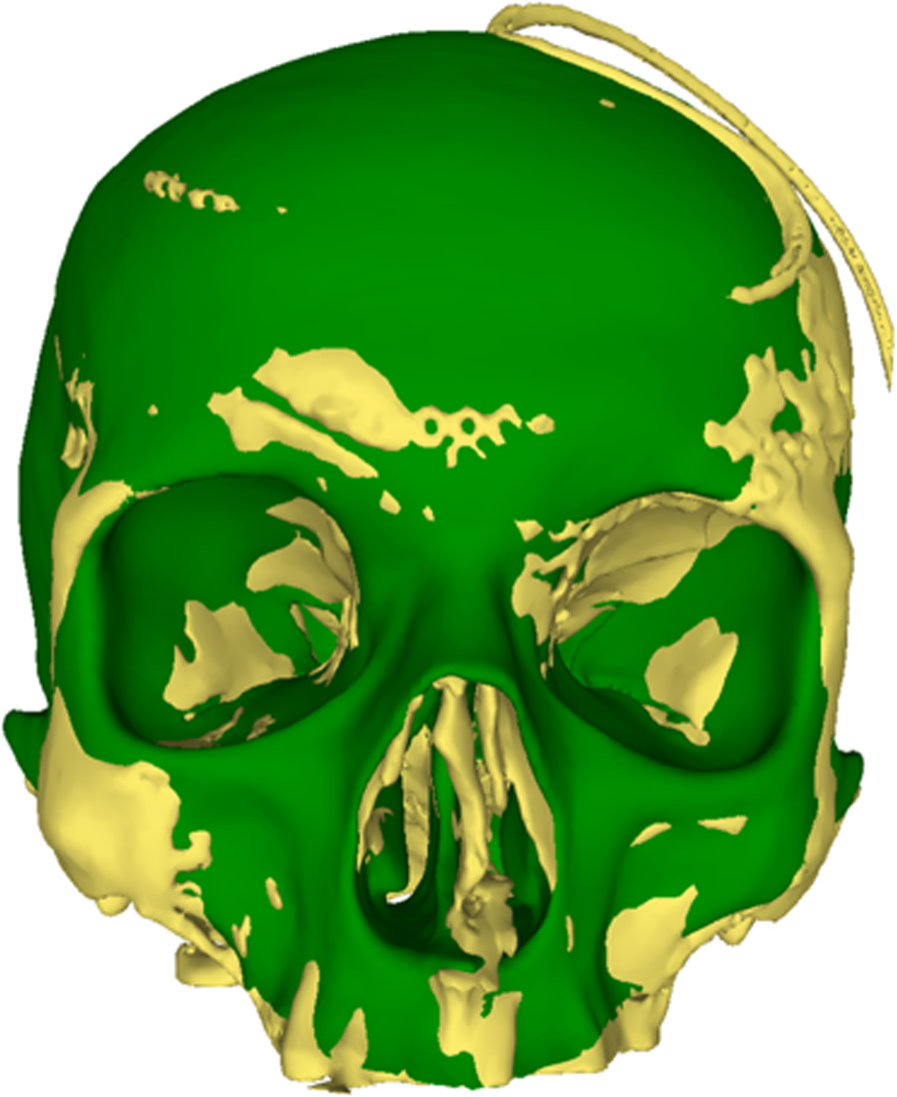
The SSM (green) is aligned on the postoperative patient skull (yellow). The changing color in the uninjured areas such as the forehead illustrates the precise fit of the SSM to the patient’s skull

The patient skull as the target surface is aligned to the gender-independent individualized shape of the SSM, followed by a rigid iterative closest point (ICP) alignment. Based on the aligned landmarks, the original SSM is then constrained to its corresponding counterparts on the aligned target, resulting in a posterior SSM. The posterior SSM only contains shapes with landmarks in the vicinity of the landmarks placed on the target mesh – allowing for isotropic variation around the landmark positions with a standard deviation (SD) of 2 mm. Starting with the mean of the posterior SSM, appropriate instances of this posterior SSM are sought, subsequently minimizing the symmetric distance to the target skull. This is done by an elastic ICP based on smoothed displacement fields. The resulting shape is a shape model instance resembling an intact cranium, very similar to the target shape (Fig. 2).

The entire processing pipeline was programmed in R [15], specifically using the R-packages Morpho, Rvcg [16], meshR and RvtkStatismo [17].

The accuracy of the resulting cranial shape was evaluated by two experienced surgeons.

**Fig. 3:**
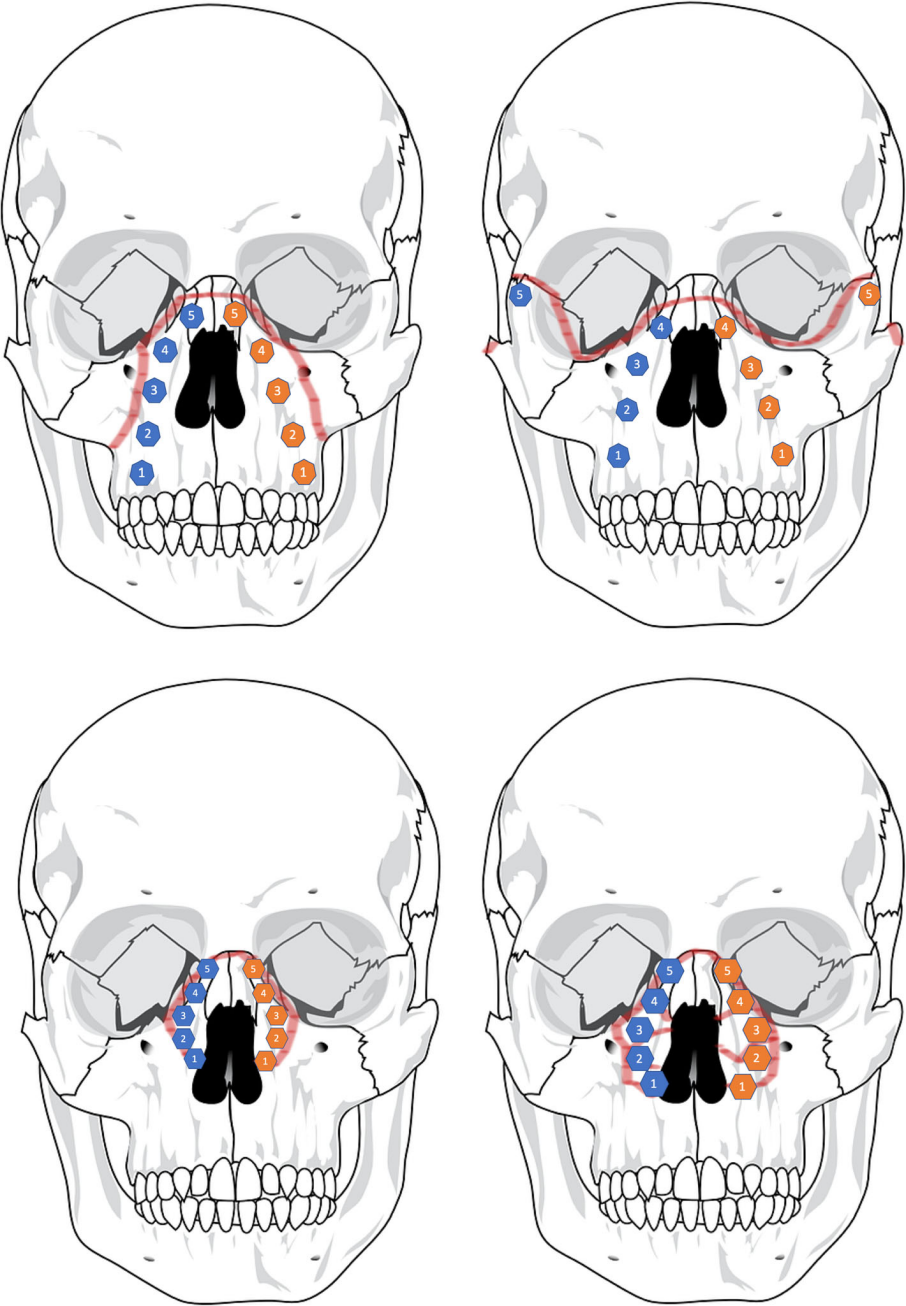
Example of the placement of deviation flags (blue and orange polygons) in Le Fort II, Le Fort III, NOE type I and NOE type II fractures for distance measurements in GOM inspect

**Fig. 4:**
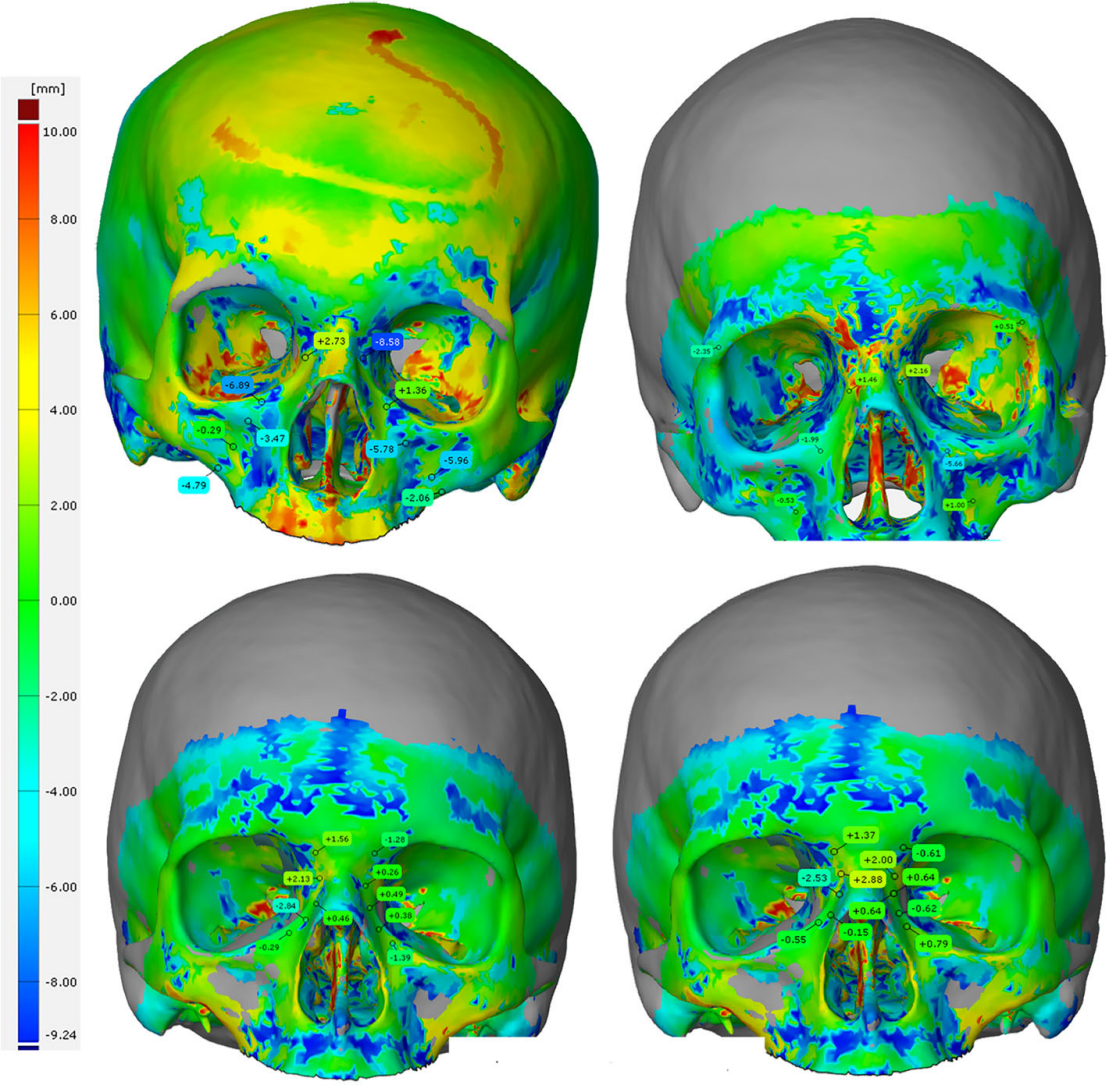
Representative images after registration of the SSM on the repositioned and osteosynthesized skulls of patients (GOM inspect with deviation flags) for Le Fort II, Le Fort III, NOE type I, and NOE type II fractures. Values with a minus stand for a still existing impact of the midface, values with a plus sign stand for protruding

The distances of the calculated ideal surface to the surgically achieved repositioning are analyzed using the GOM inspect mesh inspection software (GOM GmbH, Braunschweig, Germany). Based on the distance measurement in GOM inspect, deviation flags are placed on the medial aspect of the fracture fragment. The placement of the deviation flags corresponds to the fracture classification of the AO CMF classification and it is chosen on the medial fragment that was displaced. Placement examples are illustrated in Fig. 3. In Fig. 4, patient examples are shown within the GOM inspect software, showing the deviations in each single location.

## Results

The registration of the SSM and its evaluation could be done in all 19 cases. The automatically achieved fitting of the SSM to the unaffected bony parts was judged by the clinicians as sufficient. The midface fracture sites included are fractures of Le Fort type II (*n* = 13) and Le Fort type III (*n* = 2) as well as naso-orbito-ethmoidal (NOE) fractures type 1 (*n* = 2) and type 2 (*n* = 2).

For Le Fort type II fractures, the oral cavity, the transconjunctival and nasal approach was choosen as surgical approach in 100 % of the cases. All Le Fort III fractures were treated by a coronal, transconjunctival and oral approach. In one NOE fracture, a coronal, transconjunctival and oral approach was choosen.

For Le Fort type II fractures, the median distance between the surgically achieved repositioning result and the virtually calculated position of the midface fracture segment was –0.35 mm. Especially in the area of the medial orbital border (measurement points 4 and 5), the clinical alignment of the fragments could be done precisely; however, with regard to its suggested position, the midface complex should have been moved even further anteriorly.

The midfacial impaction of the Le Fort type III fracture was shown to be more pronounced compared to the Le Fort type II fractures. The median dorsal displacement towards the virtually calculated ideal position was –2.95 mm.

**Fig. 5:**
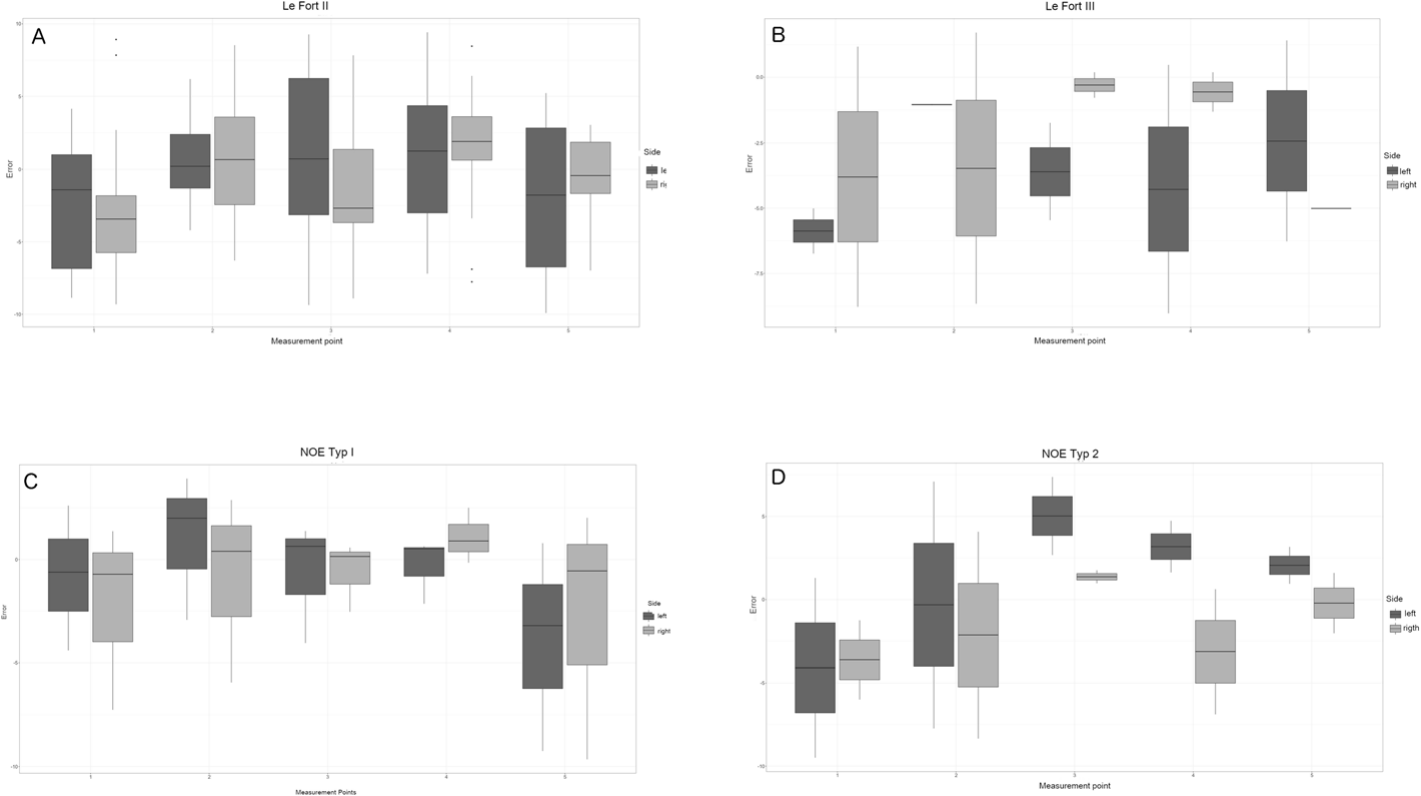
Distances in millimeters between the surgically achieved repositioning and the calculated ideal position using the SSM, shown as boxplots. The diagram shows the median and mean observations for the left and right landmark according to the placed deviation flag (Fig. 3). The lower and upper ends of the lines indicate observations outside the 9–91 percentile range. Data falling outside this range are plotted as outliers of the data. (**A**) Le Fort II fractures, (**B**) Le Fort II fractures, (**C**) NOE type 1 fractures, (**D**) NOE type 2 fractures

The median posterior displacement of the NOE complex in case of the type 1 fractures was –1.09 mm. In case of the NOE type 2, this median value was –0.27 mm. For graphical illustration, the results were displayed in four boxplots (Fig. 5) representing the different fracture types.

## Discussion

Two aspects, resulting from this study must be discussed. First, the proposed SSM could be aligned on individual fracture patterns of the midface by using landmarks on easily identifiable bony structures without manual corrections.

Second, by measure the distances between die aligned SSM and the achieved reconstruction, a clinically relevant statement could be said.

In terms of the successful application of SSM, the study proves that the technique is robust enough to virtually capture even complex fracture patterns with the precision and robustness shown in preliminary studies and to provide reconstruction suggestions [5, 13, 14].

Future research in the field of Computer-assisted surgery has to focus on the clinical feasibility of the shown technique. The SSM will be used by the surgeon to plan the fracture reduction preoperatively. Complex planning on the computer can be automated by the SSM, thus reducing on-screen time. The transfer of the SSM-based, virtually planned reduction steps into the operation room can be done by navigational devices or by intraoperative imaging modalities.

In terms of clinical message, the study confirms the known problem in complex midface trauma [9, 18]. Despite the fact that the midface is well aligned and although the classical top-to-bottom or bottom-to-top approach was respected and, in the case of our department, all fracture reductions were controlled by intraoperative CBCT, the midface still needs to be moved forward [8].

In our study, we could demonstrate that an increased fracture complexity still leads to an increased dorsal impaction, despite intraoperative imaging, especially in locations where good visibility is not guaranteed.

A special feature stands out when looking at Fig. 4. Within the orbit, larger deviations are conspicuous. One reason for this may be that in case of complex trauma at our department, the orbital floor is sometimes supplied secondarily.

For the Le Fort III fracture, the medial infraorbital rim and the lateral supraorbital rim, or in NOE fractures, the medial superior and inferior aspects of the orbital rim are more dorsally displaced than areas that can be controlled by direct visibility. The visibility is an important factor, defined by the surgical approach. The coronal approach used in the LeFort III and in one NOE fracture offers a good visibility but increases surgical invasiveness. The impaction of the midface, which is often still present, can be an argument to choose the more invasive approach more often in order to further improve the surgical result in the future.

However, if the number of patients in the individual groups is taken into account, a definitive statement regarding the absolute distance of impaction is only possible to a limited extent.

Nevertheless, to improve patient care, the clinical routine includes the option of virtual planning before the operation, using the established methods of mirroring or registration. However, the mirroring and registration techniques can only be applied to unilateral fractures [19–21]. In the case of a bilateral midface fracture, there is still no clinically proven concept of how to virtually restore the midface compartment.

## Conclusions

For the first time, the presented technique of the SSM allows the clinician to virtually reconstruct complex bilateral fractures. The postoperative virtual reconstruction can currently be used as a quality assurance measure. In the future, after further clinical evaluation, the SSM technique can be used for preoperative virtual planning, as an elementary tool of computer-assisted surgery. The automation of virtual planning reduces the time required and increases the necessary precision.

## Data Availability

The datasets used and/or analysed during the current study are available from the corresponding author on reasonable request.
