# Correction to: The statistical shape model as a quality assurance measure in the treatment of complex midface fractures: a case control study

**DOI:** 10.1186/s13005-022-00306-5

**Published:** 2022-01-26

**Authors:** Marc Anton Fuessinger, Steffen Schwarz, Mathieu Gass, Philipp Poxleitner, Leonard Simon Brandenburg, Stefan Schlager, Marc Christian Metzger

**Affiliations:** 1 Department of Oral and Maxillofacial Surgery, Albert-Ludwigs University Freiburg, Hugstetterstr. 55, 79106 Freiburg, Germany; 2 Department of Physical Anthropology, Albert-Ludwigs University Freiburg, Hebelstr. 29, 79104 Freiburg, Germany

**Correction to: Head Face Med 17, 44 (2021).**

**https://doi.org/10.1186/s13005-021-00296-w**

Following the publication of the original article [1], authors spotted error on one of the author names. Leonard Brandenburg should be: (First name) Leonard (Middle name) Simon (Family name) Brandenburg. The correct name is shown above in the author group section.
